# Aesthetic Rehabilitation of Anterior Teeth With Molar Incisor Hypomineralization—A Case Report of a New Approach to Selective Removal

**DOI:** 10.1002/ccr3.72872

**Published:** 2026-06-05

**Authors:** Mohemed‐Salim Doueiri, Sakuntha Ratnapreya, Roland Frankenberger

**Affiliations:** ^1^ MIH‐Zentrum‐Berlin Berlin Germany; ^2^ Department of Pediatric Dentistry University Medicine Greifswald Greifswald Germany; ^3^ Department of Operative Dentistry and Endodontics, Dental School University of Marburg Marburg Germany

**Keywords:** dental aesthetics, molar incisor hypomineralization, resin composites, selective removal

## Abstract

Molar incisor hypomineralization (MIH) is a qualitative enamel defect that can affect not only molars but also anterior teeth leading to both pain and aesthetic problems. There are various treatment options for MIH such as resin infiltration, microabrasion, and resin composite restoration. A 14‐year‐old girl presented with opaque MIH lesions on upper central incisors. The hypomineralized tissue was selectively removed using only Soflex discs and white stone burs, and subsequently restored with resin composite. The treatment preserves healthy tooth structure while simultaneously removing completely the affected portion of the tooth painlessly. The final result was aesthetically pleasing and the technique proved to be an easily reproducible treatment option for anterior teeth severely affected with MIH.

## Key Clinical Message

The aesthetic rehabilitation of teeth severely affected by molar incisor hypomineralization (MIH) begins with the appropriate removal of affected tissue. Abrasive polishing discs and white stones traditionally used for polishing restorations, are able to selectively remove the weakened MIH affected enamel. This technique is an easily reproducible technique for general dentists producing excellent aesthetic results.

## 1 Introduction

Molar incisor hypomineralization (MIH) is a qualitative dental enamel disorder occurring during late amelogenesis. It manifests in at least one permanent first molar and may also affect permanent incisors. Affected anterior teeth often exhibit opaque discolouration ranging from white to brown, associated with weakened chalky enamel [1]. This disease also presents an aesthetic challenge with “unsightly” patches on labial surfaces of anterior teeth.

The literature in the field enlists various treatment modalities for MIH affected anterior teeth. Resin infiltration is a popular treatment modality with a significant reduction in post eruptive breakdown (PEB). However it is most often successful with mild to moderate cases of MIH [2]. Wong and Winter investigated the success of micro abrasion with abrasive paste and 18% hydrochloric acid for the treatment of anterior opacities. Their results were acceptable in single line/patched defects. However, it was not the same with multi‐line/diffused defects [3]. Vital bleaching is another treatment option but is not viable for advanced cases with severely weakened enamel [4].

Removal of affected enamel with a bur and restoration with resin composite addresses the aesthetic problem at the cost of extensive preparation. We believe this case report of selective removal of affected enamel tissue would enable clinicians to be conservative during preparation while achieving an excellent aesthetic outcome.

## 2 Case History and Examination

**FIGURE 1:**
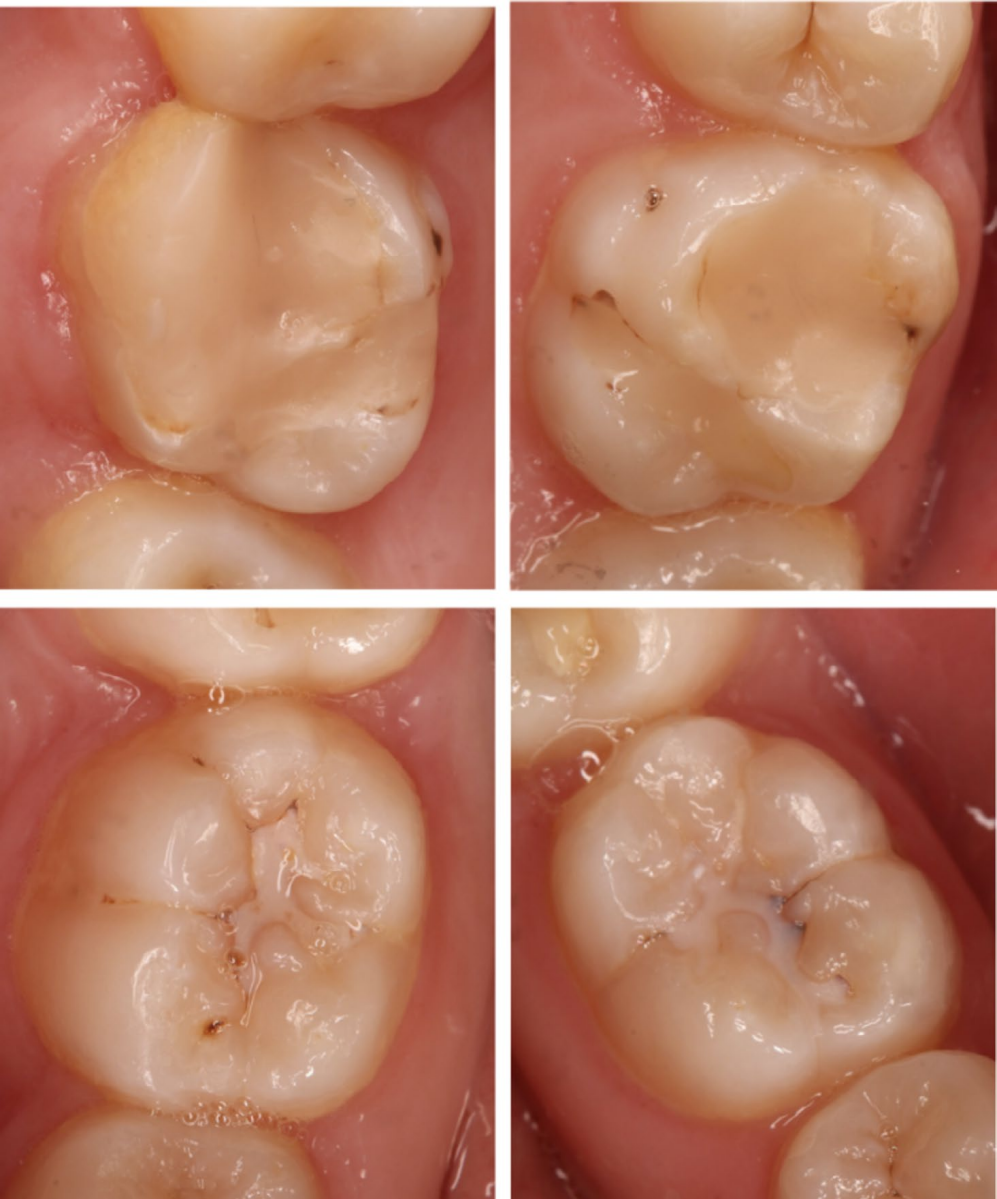
First permanent molars affected by MIH.

**FIGURE 2:**
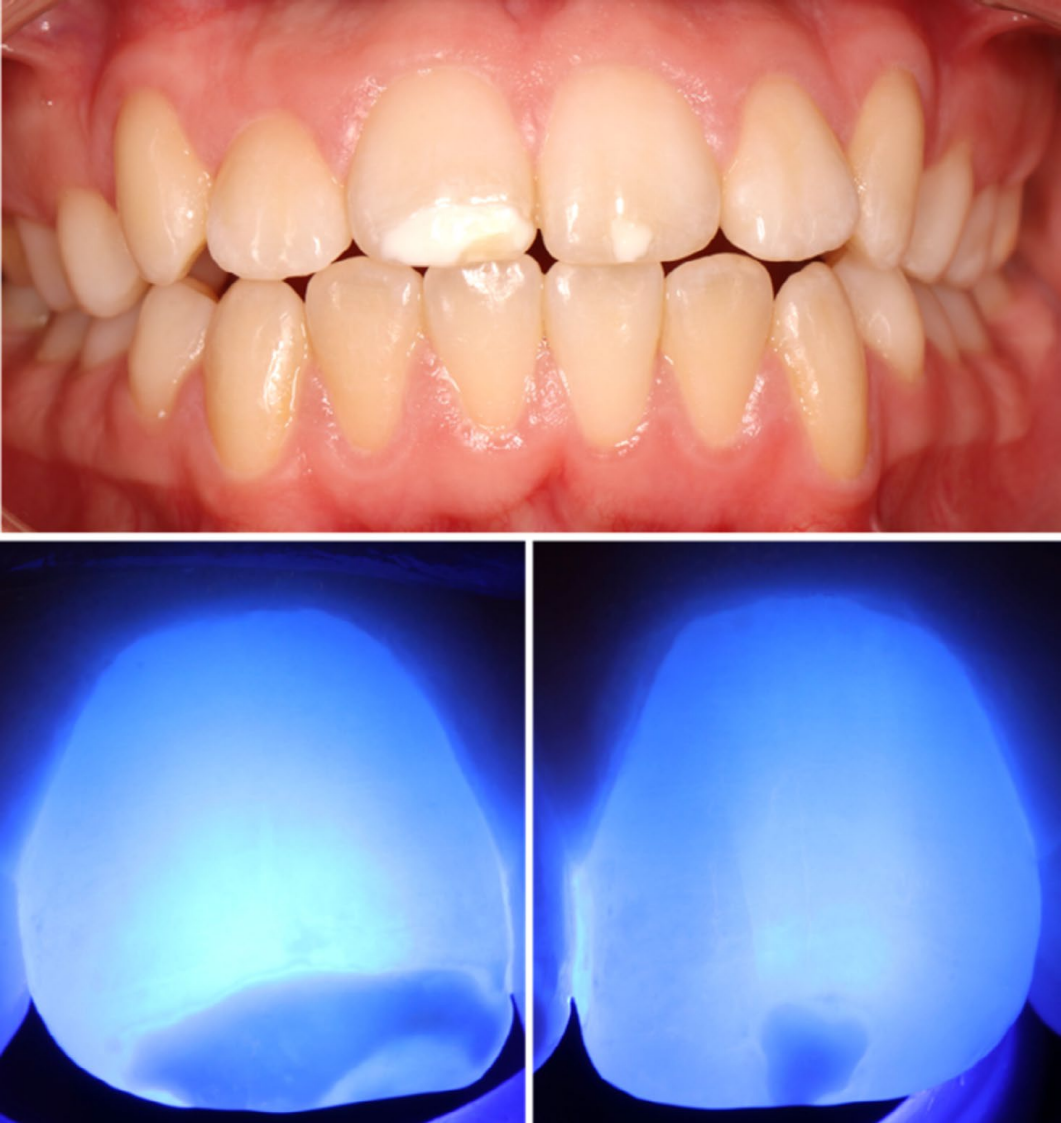
Preoperative anterior view with MIH opacities on 11, 21, and preoperative transillumination of 11 and 21.

A 14‐year‐old female patient with no relevant medical history presented to a pediatric dental practice in Berlin, Germany with opaque lesions on 11 and 21. Intraoral examination also revealed an atypical resin composite restoration with occlusal, buccal, palatal and mesial extensions with 16, a large occlusal resin composite restoration with 26 and hypomineralized opacities occlusally with 36 and 46 (Figure 1). Both depth and extent of the opacities with 11 and 21 were assessed using transillumination using a light curing lamp (D‐Light, GC Europe, Leuven, Belgium/high‐power mode) (Figure 2). The opacities were clearly visible during speaking and smiling, leading to reduced self‐esteem and psychosocial stress. The patient and her mother expressed a clear desire for prompt aesthetic rehabilitation.

## 3 Differential Diagnosis, Investigations, and Treatment

The opaque lesions were situated in the incisal 1/3 of 11 and 21. There was neither PEB nor dentinal hypersensitivity present. No additional investigations were required. The treatment options including resin infiltration, microabrasion followed by resin infiltration and selective removal of affected enamel tissue with resin composite rehabilitation were offered to the parent and patient. The parent and patient were unsure about the predictability of the outcome with resin infiltration and also found it to be expensive. Therefore they decided to opt for the third treatment option presented to them, with the expectation of an excellent aesthetic outcome. The aim was to remove the discolored, hypomineralized enamel while preserving the healthy tooth substance and to aesthetically restore the resulting defects. The authors wish to present the detailed steps in preparation and restoration of 11.

**FIGURE 3:**
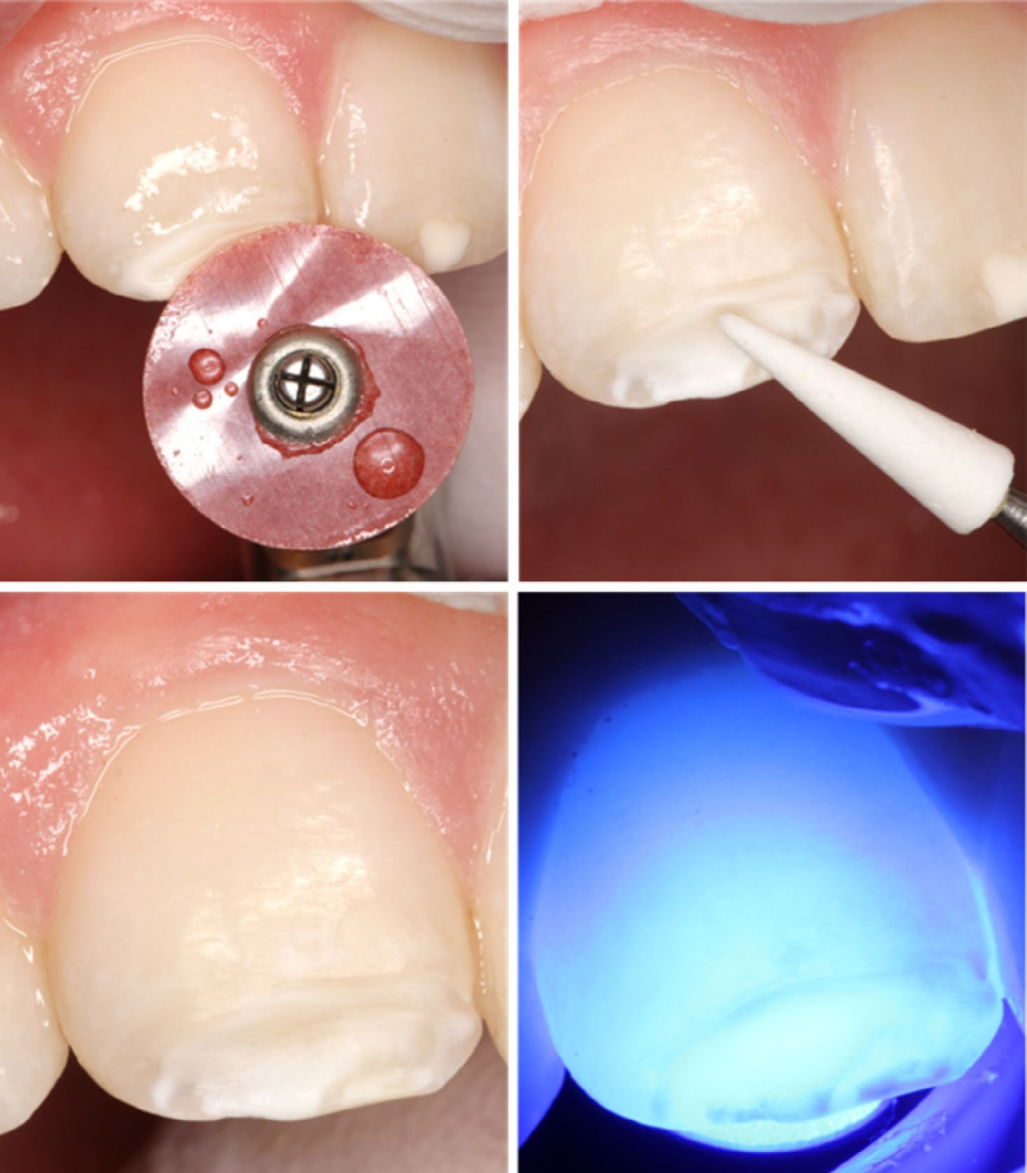
Selective preparation of 11 using Soflex disc (coarse) and white stone, with extent of removal controlled by transillumination.

Soflex discs (3M) (coarse) and white stone burs (MD 661XF 025, Hager&Meisinger, Neuss, Germany) were used to selectively remove the affected enamel in 11. Rotary instruments were used at 10,000–20,000 rpm with copious water cooling. The extent of removal was controlled by visual examination and transillumination (Figure 3).

**FIGURE 4:**
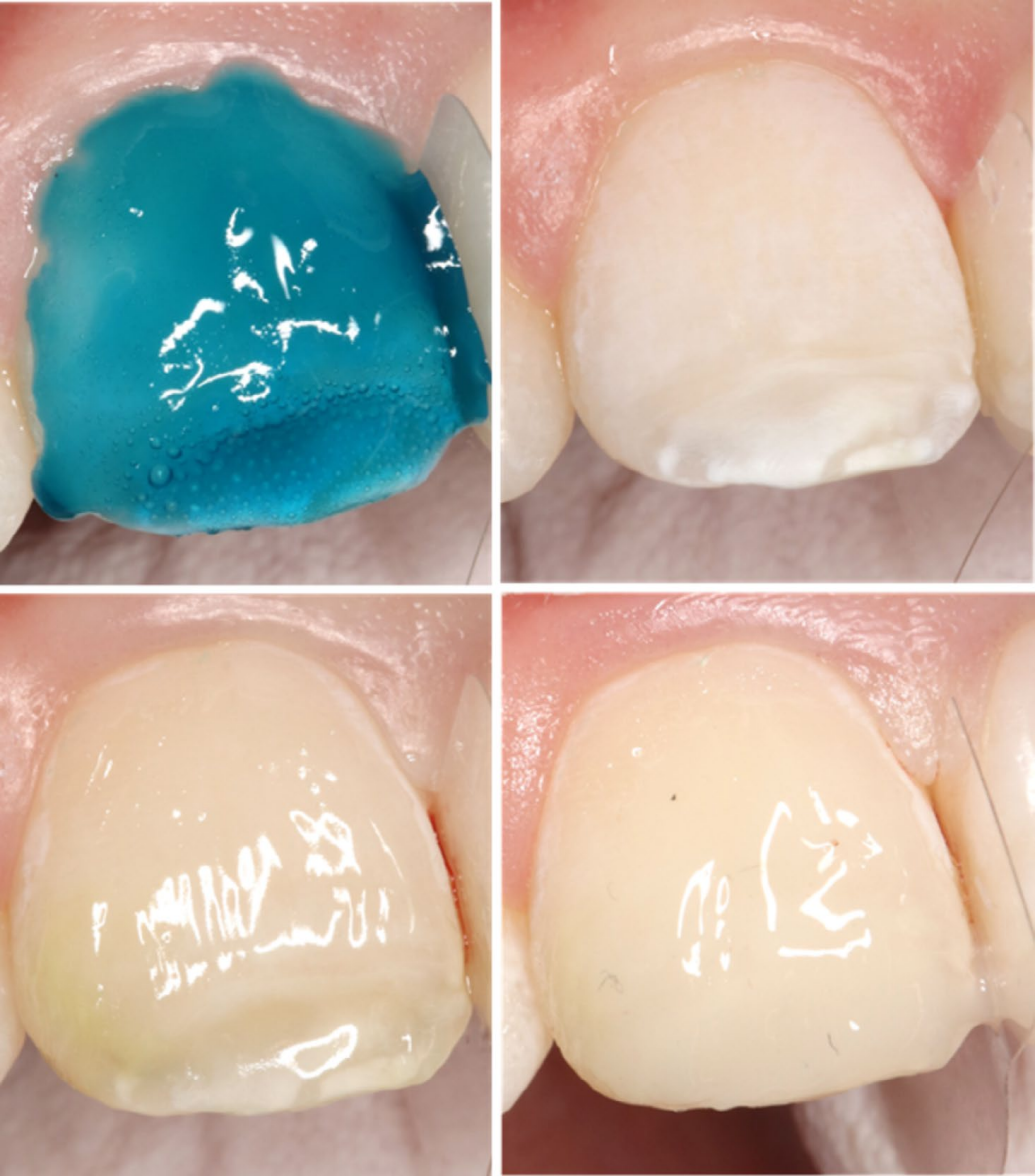
Steps in Resin Composite Restoration of 11.

Etching was performed with Super Etch 37.5% phosphoric acid gel (SDI, Melbourne, Australia). Following thorough rinsing of the etchant and drying with a jet air spray, an adhesive system (ZipBond, SDI) was applied, agitated on the dentinal surfaces for 15 s, and light cured for 10 s. Resin composite was applied step by step, initially a thin layer of flowable resin composite (Ceram.X Spectra ST Flow A1, Dentsply Sirona) was applied and light cured. This was followed by a fine layer of opaque resin composite (Luna 2 OA1/OA2, SDI) to mask the underlying affected dentin. Finally, a layer of enamel flowable resin composite (Aura EasyFlow AE1, SDI) was applied (Figure 4). Polishing was performed with the Soflex polishing system (medium to superfine) to high gloss.

## 4 Conclusion and Results

**FIGURE 5:**
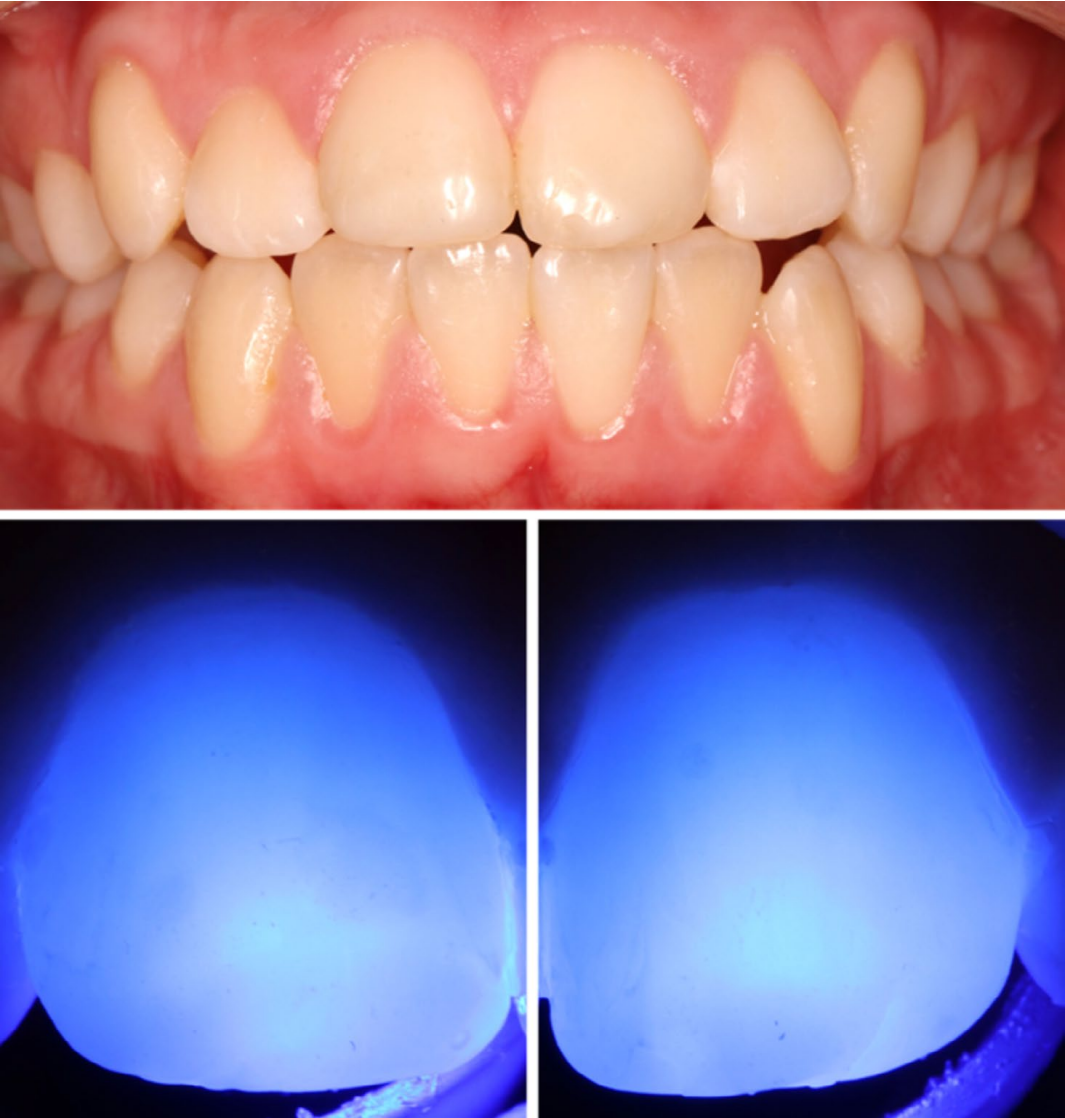
Postoperative anterior view after 6 months and postoperative transillumination of 11 and 21.

At the follow‐up examination after 6 months, the opacities were no longer clinically visible and also unnoticeable with transillumination (Figure 5). The patient and parents were extremely satisfied with the result. A semi‐annual follow‐up was recommended.

## 5 Discussion

The hypomineralized enamel contains reduced calcium and phosphorous, lower hardness and greater porosity in comparison to healthy enamel [5]. This greatly increases the risk of developing caries and undergoing PEB. Furthermore the altered structure of the enamel makes the treatment of MIH related anterior tooth opacities with traditional methods, challenging.

Literature shows promising results with resin infiltration. The findings from a systematic review concluded that resin infiltration was effective in improving color and optics in comparison to minimally invasive treatments and fluoride varnish application in MIH affected teeth [2]. Furthermore, Casaña‐Ruiz et al., reported significant improvements in lesion size, color parameters, sensitivity and aesthetic perception with using resin infiltration on MIH affected anterior teeth in a population of children and adolescents [6]. However, Crombie et al. reported that there was an increase in Vickers microhardness, in only the superficial areas of MIH affected enamel where the resin was able to infiltrate [7]. Furthermore, it was also reported that the sound enamel was thickly and evenly infiltrated as opposed to the MIH affected enamel. Larger yellow brown lesions which are located deeper in the enamel, require a more invasive approach while preserving the integrity of the healthy underlying enamel.

Additionally, literature states that unlike fluorotic lesions, MIH lesions form an acute angle with the tooth surface making resin penetration problematic [8]. Attal et al. proposed the technique of “deep infiltration” to solve this problem by removing the “ceiling” of the lesion followed by resin infiltration. However the authors of this technique accepted that if the defect left by removal was significant, it should be restored with resin composite [9]. On the other hand, in the technique described in the above case report, the clinician is able to control the removal of affected enamel using visual examination and transillumination. Gharavi et al., reviewed the literature on and reported a successful case of the Etch–Bleach–Seal technique which combined the ability of phosphoric acid to condition enamel crystals, the chemical oxidation of sodium hypochlorite and the sealing ability of resin infiltration [10]. However there is limited evidence on this technique and the possible long term implications of using sodium hypochlorite in this clinical scenario.

Microabrasion and vital bleaching have shown success in the management of small anterior teeth lesions [3, 4]. However, with microabrasion it is challenging to selectively remove the affected enamel. Moreover, lesions treated by microabrasion have a rougher surface and are more prone to staining [11]. Due to these limitations, some clinicians have combined in‐office bleaching, microabrasion and resin infiltration to achieve better aesthetic results [12]. Therefore in the above case report, the treatment option of microabrasion was offered, albeit in combination with resin infiltration.

The technique described in this case report, exploits the reduced strength of the MIH affected portion of the enamel. This ensures selective removal of affected enamel. The abrasive polishing discs and white stones are traditionally used to polish resin composite restorations without any harm to the healthy tooth structure. Once the tooth is prepared, the remaining procedure is identical to a routine anterior resin composite restoration. This makes this technique low in technique sensitivity and easy to replicate for general dentists. Additionally, this pediatric patient “friendly” technique gave an excellent aesthetic outcome without the need for local anesthesia.

However, a limitation of this technique could be the limited applicability in narrower or cervical anterior tooth MIH lesions where the diameter and the angle of approach of the Soflex disc may limit selective removal. It is important to note that, rubber dam isolation was not used in this case as cotton roll isolation was sufficient in achieving moisture control. Nonetheless, further studies and clinical trials are required to systematically evaluate this treatment modality.

## Author Contributions

**Mohemed‐Salim Doueiri:** conceptualization, investigation, methodology, project administration, visualization, writing – original draft, writing – review and editing. **Sakuntha Ratnapreya:** conceptualization, writing – original draft, writing – review and editing. **Roland Frankenberger:** project administration, supervision, writing – original draft, writing – review and editing.

## Funding

The authors have nothing to report.

## Consent

Written informed consent was obtained from the patient and her legal guardian for publication of this case and accompanying images.

## Conflicts of Interest

The authors declare no conflicts of interest.

## Data Availability

The data that support the findings of this study are available from the corresponding author upon reasonable request.
